# Physical activity and motor skills in children attending 43 preschools: a cross-sectional study

**DOI:** 10.1186/1471-2431-14-229

**Published:** 2014-09-12

**Authors:** Line Grønholt Olesen, Peter Lund Kristensen, Mathias Ried-Larsen, Anders Grøntved, Karsten Froberg

**Affiliations:** Centre of Research in Childhood Health (RICH), Department of Sports Science and Clinical Biomechanics, University of Southern Denmark, Campusvej 55, 5230 Odense M, Denmark

**Keywords:** Accelerometer, Cluster Analysis, Intraclass Correlation, MABC-2, KTK test

## Abstract

**Background:**

Little is known about health characteristics and the physical activity (PA) patterns in children attending preschools. The objective of this study was to describe the gender differences in relation to body mass index (BMI), motor skills (MS) and PA, including PA patterns by the day type and time of day. Additionally, the between-preschool variation in mean PA was estimated using the intraclass correlation.

**Methods:**

We invited 627 children 5–6 years of age attending 43 randomly selected preschools in Odense, Denmark. Aiming and catching MS was assessed using subtests of the Movement Assessment Battery for Children (Second Edition) and motor coordination MS was assessed by the Kiphard-Schilling body coordination test, Körperkoordination Test für Kinder. PA was measured using accelerometry. The PA patterns were analysed using mixed models.

**Results:**

No gender differences in the BMI or norm-referenced MS risk classification, or the average weekly PA level or patterns of PA were observed. However, boys performed better in the aiming and catching score (p < 0.01) and in the motor coordination score (p < 0.05) on average. Girls performed better in the balance subtest (p < 0.001). Relative to the norm-referenced classification of MS, the Danish sample distribution was significantly well for aiming and catching but poorer for the motor coordination test.

The total sample and the least active children were most active on weekdays, during preschool time and in the late afternoon at the weekend. However, a relatively larger decrease in PA from preschool to weekday leisure time was observed in children in the lowest PA quartile compared to children in the highest PA quartile. Finally, the preschool accounted for 19% of the total variance in PA, with significant gender differences.

**Conclusions:**

Results of this study could provide a valuable reference material for studies monitoring future trends in obesity, MS and PA behaviour in Denmark and other countries.

Knowledge about sources of variation in PA among preschool children is scarce and our findings need to be replicated in future studies. A potentially important finding is the large between-preschool variation in PA, indicating that especially girls are very susceptible to the environment offered for PA during preschool attendance.

## Background

There is increasing evidence of a positive link between health outcomes such as obesity, motor skills (MS) and physical activity (PA) in preschool-aged children [1]. Even so, a large proportion of preschool children are not sufficiently physically active [2], even during preschool attendance [3, 4].

In Denmark, 97% of all children aged 3–5 years old [5] spend a high proportion of their waking hours at preschool, often also referred to as a kindergarten. The preschools are placed within the school districts but are not an integrated part of the schools. The day-to-day pedagogical practice is characterised by engagement in structured activities and unpredictability. Furthermore, focus is on child-to-child relations, dialogue and embodiment, and also on outdoor time, as participating in both calm and vigorous activities is of high priority [6]. In the Danish preschools, there is equal focus on children’s independent play, planned learning by activities and practicing everyday life [6, 7]. Since 2004, the aspect of learning through theory-based curricular strategies (learning plans) has been signed into the Danish legislation [6], and motor development and physical activity have been topics of focus.

A potentially beneficial effect of the Danish preschool system could be higher PA levels among Danish children compared to children experiencing sedentary pre-academic activities during preschool attendance [8–10]. In earlier studies, the preschool attended has been shown to be a strong predictor of children’s PA behaviour [11, 12]; however, this potentially important finding needs to be confirmed in a larger sample of preschools.

In studies of children’s PA behaviour, divisions between preschool time, weekday and weekend day leisure PA behaviour is considered important, as preschool children’s PA behaviour is influenced by different factors in different settings [13]. So far, only a few studies have provided information on preschool children’s PA level across day types, time of day, and preschool and leisure settings [10, 14–16]. The results of these studies could be significantly influenced by sociocultural factors such as cross-cultural variations in the organization of a preschool day [8–10], and different attitudes and expectations about children’s cross-gender behaviour [17].

Motor coordination has also been reported to be positively associated with preschool children’s PA behaviour [18]. However, few studies in Europe have reported the prevalence of movement difficulties relative to norm-referenced data in a high number of apparently healthy preschool children with respect to object control skills such as aiming and catching [19] and motor coordination [20–24].

The present explorative study included data from 43 randomly selected preschools in Denmark. The study aimed to:

1. Describe the characteristics of the study population in relation to BMI, MS and PA by gender.
2. Investigate whether PA differs according to the day of the week and across gender.
3. Investigate whether PA during preschool and daytime leisure periods differs between weekdays and weekend days and across gender for the total population of children, as well as for children with low levels of PA.
4. Investigate whether relative changes in PA from preschool to leisure time and from weekday to weekend days, respectively, differs across children in different PA quartiles.
5. Determine the extent to which PA levels vary between preschools, and estimate whether or not the variation is gender dependent.

The results of this study could have important methodological implications for the design of future cluster randomized trials in this population, both in terms of estimating required sample sizes and assessing the need to adjust for temporal variations in PA. Furthermore, a good reference material for the present population is important when monitoring future trends in obesity, MS and PA behaviour in Denmark and other countries.

## Methods

### Study design

The study is a cross-sectional study.

#### Selection of preschools

The sampling frame was a complete list of all traditional public preschools in the municipality of Odense, Denmark. Sample size estimations were determined in consultation with a statistician and based on data from an earlier Danish preschool study [11]. All preschools (n = 117) were stratified according to location (urban or rural), socioeconomic status in the school district and the size of the preschool. The preschool size was determined based on information from the Odense municipality, and was defined as the weighted sum (1:1) of total indoor area (m^2^ per child) and the total accessible outdoor area (m^2^). To be able to investigate the importance of PA in relation to location, it was necessary to oversample slightly in rural areas. In total, 43 preschools (13 rural preschools) were randomly selected, and data were collected from March to July 2009.

#### Selection of children

In the selected preschools, all apparently healthy children (n = 627) born in the year 2003 (5–6 years of age) were invited to participate in the study. Children who were no longer enrolled in the preschool by the start of the data collection were excluded from the study.

#### Ethics

The Regional Scientific Ethical Committee in Southern Denmark reviewed the application for ethical approval for this study, and concluded that formal ethics approval was not required. This conclusion was drawn based on the observational nature of the study and that the measurements carried out were noninvasive and not in any other way assessed to be harmful to the children (project ID:S-20080093). Parents of the participating children received a passive informed consent form that explained the nature and procedures of the study and if parents and/or their child(ren) did not want to participate, they could withdraw during any stage.

### Anthropometry

Body height was measured without shoes to the nearest 0.5 cm using the Harpenden stadiometer (West Sussex, UK). Body mass was measured wearing underwear to the nearest 0.1 kg using an electronic scale (Seca 882, Brooklyn, NY). The proportion of children being underweight, normal or minimum overweight were based on well-established cut-points according to BMI [25, 26].

### Movement skills

Subtests from the English Movement Assessment Battery for Children (Second Edition) (MABC-2), age band 1 (3–6 years) were selected to evaluate static balance, fine MS, and aiming and catching MS; the latter two subtests also provided a component aiming and catching score [27].

The German Kiphard-Schilling body coordination test (5–14 years), Körperkoordination Test für Kinder (KTK) [28], was selected to evaluate motor coordination, specifically dynamic balance. Both tests show good reliability and validity [29, 30].

The MS tests and anthropometrics were carried out at the preschools before noon. The preschool children were tested for 20–25 minutes, two children at a time, and by the same two trained observers. The reproducibility (test-retest reliability) for each norm-referenced subtest was estimated and presented by the intraclass correlation coefficient (ICC) in 50 randomly selected children (46% boys). Retests were carried out 0.25–4 days after the initial test.

The first trained observer assessed anthropometrics and the four product-oriented subtests from the MABC-2 test: coins in a box (piggy bank) (ICC = 0.60); catching a beenbag and throwing a beanbag into a target (ICC = 0.58); and one-legged balance (ICC = 0.78). The second trained observer carried out the KTK test: walking backwards on balance beams of decreasing width (6 cm, 4.5 cm, 3 cm) (ICC = 0.77); jumping on one leg at a time over an increasing number of foam blocks (length: 50 cm*width; 20 cm*height; 5 cm) (ICC = 0.89); jumping laterally with feet together over a small beam (60 cm*4 cm*2 cm) for 15 seconds (ICC = 0.83); and moving sideways, shifting between two platforms (25 cm*25 cm*2 cm, supported on four legs 3.7 cm high) for 20 seconds (ICC = 0.83). ICC for the total KTK score was 0.89.

#### Motor skill outcome

A test score was recorded for each subtest, and then converted into standard scores based on age-specific norm-referenced data for the MABC-2 (mean/SD 10 ± 3) and KTK (mean/SD 100 ± 15) test, respectively. The prevalence of being categorized as at-risk or with movement difficulties correspond to a cut-point of ≤1 standard deviation and ≤2 standard deviations below the mean age-specific norm-referenced standard score, respectively.

### Physical activity

The children’s PA levels were assessed using ActiGraph’s activity monitors (GT1M version 4, and GT3X, Pensacola FL.) on 5–6 weekdays and 2 weekend days. PA data collection occurred during a period of 4 weeks in May and June. When using the GT1M and GT3X generations of ActiGraph’s activity monitors simultaneously, activity counts seem, with the exception of one study [31], to be comparable if activity data from vertical axis is used [32–34]. The activity monitor was worn on the right hip, close to the skin, except when sleeping or showering. To minimize reactivity in the assessment, the activity monitors did not start to record PA data until the following morning of delivery. The preschool staff kept a daily record of each child’s arrival and departure times.

#### Physical activity data reduction

PA data were collected with 1 or 5 seconds/epoch. Inclusion criteria were three valid days of at least 10 hours measurement, including at least one weekend day. Zero activity periods of 60 minutes were interpreted as “activity monitor not worn”, and therefore removed from the summation of activity. Weekdays on which a child was sick were excluded from the analyses, since these days did not represent a typical day. All holidays (n = 74) were included as Sunday.

To categorize children in groups of different PA levels, the weekly PA level was divided into quartiles of PA based on gender-specific cut-points. Custom-made software was used for data reduction.

#### Physical activity outcome

PA was presented as mean counts per minute. In case a difference in the PA level between weekdays and weekend days is present, the weekly PA level representing a whole week will be calculated as the average weekday and weekend PA weighted by 5/7 and 2/7, respectively.

### Sociocultural factors

The country of birth of the child’s mother was assessed by a parental questionnaire answered during PA data collection and based on information from the preschools, and subsequently categorized into three groups according to their country of origin: Denmark; other Western countries; or non-Western countries.

The parental education level was based on the Danish educational nomenclature (DUN) (2006) developed by Statistics Denmark [35]. The mean of the two parents’ DUN level (1–9) was categorized into low (DUN level 1 to <4), middle (DUN 4 to <6) or high (DUN 6–9). If information was given from one parent only – as was the case for 15% of the children – a single DUN level was used.

### Statistics

The data were analysed in STATA 12 (Stata Corp, College Station, TX), with a significance level of P < 0.05. Inverse probability weighting was used in all descriptive and statistical analyses to adjust for oversampling. Before taking the inverse, the sampling weight was calculated as the sampling frequency of each specific combination of the stratifying parameters (SES, size and location) divided by the total number of preschools in the Odense municipality with that specific combination.

Descriptive statistics for the participants’ characteristics are displayed as means (SD)/median (5^th^–95^th^ percentile). Pearson’s chi-squared test was used to compare proportions of groups, while t-tests compared weighted means between genders.

The differences in the PA level between days of the week, weekend versus weekdays, and during different time periods of the day were analysed using mixed models, with the preschool and the individual child treated as random effects. Gender separated models were reported after being adjusted for the country of birth of the child’s mother and the mean parental educational level; the latter was tested insignificantly in all models (p > 0.05) and left out in the final models in order to preserve statistical power. However, the estimated effect did not change when this was excluded, but increased the number of participating boys (girls) from 167 (169) to 192 (194). All models were tested for an interaction with gender after being adjusted for the country of birth of the child’s mother (n = 386).

Similar models were run for children in the low PA quartile (n = 94) after adjusting for gender. Furthermore, the relative difference (%) in PA between the preschool and leisure time setting on weekdays, as well as the difference in PA between weekdays and weekend days, were studied across quartiles of weekly PA. Wald tests were applied, and adjusted means with 95% CI were presented.

The between-preschool variation in mean PA during preschool attendance was calculated without any adjustments for independent variables, and represented by the intraclass correlation (ICC) [36]. The 95% CI of the ICC was computed using the delta method.

## Results

### Preschools and sample

All 43 preschools participated in the study. In accordance with the exclusion criteria, children leaving preschool before the data collection (n = 20) were excluded from the study.

In total, 607 out of 627 invited children were eligible to participate in the study. The mean age (SD) of these children was 5.8 (0.3) years, of which 299 (50%) were boys. The mean BMI was 15.6 (1.4) kg\m^2^ (range: 12.8–21.7 kg\m^2^). No gender differences were identified for age, height, body weight and BMI (data not shown).

The BMI, MS, PA and the parent questionnaire response rate were 93%, 93%, 64% and 78%, respectively. Reasons for non-participation in the assessment of MS and BMI were mainly due to casual absences, such as holiday or sick leave, and families deciding not to participate in the study (n = 10).

A test for possible selection bias due to missing information on PA showed that among children without valid PA data (n = 221), there was a higher weighted proportion of mother’s born outside (non-western countries) of Denmark (23% versus 9%, p < 0.001), of parents with low mean DUN level (33% versus 16%, p < 0.001) and children categorized as obese (4% versus 1%, p < 0.05) according to Cole et al. [25, 26], compared with the group of children with valid PA data (n = 386). No difference between groups of children with or without valid PA data was found for age, gender, height, KTK (classification), aiming and catching component score, location or preschool size.

### Body weight classification and movement skills

Irrespective of gender, the prevalence of being classified as underweight (grade 2), underweight (grade 1), overweight or obese in the 567 children was 1%, 7%, 9% and 2%, respectively, according to Cole et al. [25, 26].

Table 1 describes the preschool children’s raw and mean norm-referenced scores for the MABC-2 and the KTK tests by gender. Compared to the average original norm-referenced scores for MS, Danish boys and girls scored higher than average on the MABC-2 tests, but lower in the KTK subtest (except walking backwards) as well as the total KTK score. We tested for gender differences in the mean norm-referenced score in selected MABC-2 tests and the KTK test. Boys had a higher mean norm-referenced score according to the MABC-2 aiming and catching component score (p < 0.01) and the KTK test (p < 0.05). Girls performed better in the MABC-2 balance subtest (p < 0.001). There was no difference found between the two genders in the MABC-2 manual dexterity subtest.

**Table 1:** **Movement skill performance in 5-6 year old preschool children**

	Girls		Boys		T-test
	N	Mean (SD)	N	Mean (SD)	p-value
**Total**	308		299		
**MABC-2** ^**a**^ **selected subtest**					
Manual dexterity					
Coins in box, preferred hand, sec	285	17.8 (2.2)	281	18.4(2.4)	
Coins in a box, other hand, sec	284	19.9 (3.0)	281	20.2(3.3)	
Total coins in a box, norm score^c^	284	10.8 (2.2)	281	10.4 (2.4)	0.10
Aiming and catching					
Catch a beanbag, n	285	7.4 (2.1)	281	7.6(2.1)	
Throw a beanbag onto mat, n	285	6.1 (1.9)	281	6.7(2.0)	
Total aiming and catching, norm score^c^	285	10.6 (2.7)	281	11.3 (2.9)	<0.01
Balance skills					
Balance preferred leg, sec	284	26.2(7.1)	281	23.4(8.7)	
Balance other leg, sec	284	21.9(9.3)	281	18.4(10)	
Total balance on one leg, norm score^c^	284	12.1(2.5)	281	11.2(2.8)	<0.001
**KTK** ^**b**^ **all subtest**					
Motor coordination					
Walking backwards, steps	284	33.5 (14.3)	280	30 (14)	
Walking backwards, norm score	284	104.0 (16.7)	280	100.2 (16.1)	
Hopping on one leg, points	284	15.6 (9.2)	281	14.2 (10.3)	
Hopping on one leg, norm score	284	86.0 (14.1)	281	96.6 (14.1)	
Jumping sideways, jumps	284	27.9 (8.6)	281	26.8 (8.2)	
Jumping sideways, norm score	284	95.2 (14.5)	281	98.7 (15.9)	
Moving sideways, shifts n	284	28.1 (5.4)	281	27.8 (6.1)	
Moving sideways, norm score	284	98.0 (12.3)	281	97.5 (13.3)	
Total KTK, sum of raw scores	284	105.2 (30.0)	280	99.0 (31.4)	
Total KTK, norm score^c^	284	94.4 (14.8)	280	97.7 (14.9)	<0.05

^a^Selected subtests from the Movement Assessment Battery for Children (MABC-2).

^b^Körper Koordinationstest für Kindern (KTK).

^c^Test for gender differences in selected MS tests.

For each test raw test scores (sec, n, steps, points, jumps, shifts) and calculated norm-referenced scores (mean (SD)) being 10(3) for MABC-2 and 100(15) for KTK are presented.

T-test tested for gender differences in the mean norm-referenced score in selected MABC-2 tests and the KTK test.

No gender difference was observed in the MABC-2 aiming and catching component score (p = 0.07) or the KTK score (p = 0.18) when participants were classified as having either difficulty, being at risk, normal, or having good or high motor performance relative to the original norm-referenced MS risk-classification cut-points.

**Figure 1:**
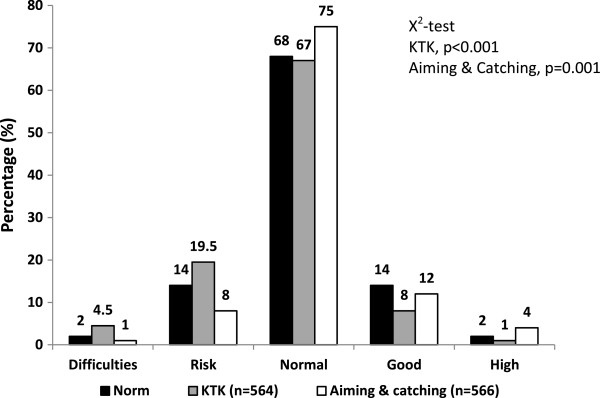
**Distribution of expected and observed norm-referenced motor skill classification in 5-6 year old children.** The motor skill categories are based on the Körperkoordination Test für Kinder (KTK) (1974), and the MABC-2 aiming and catching component score (2005). The figure illustrates a comparison of the expected original norm-referenced motor skill risk-classification (black bars) with the observed Danish sample expressed in percentages. The figure includes data from 563 children 5-6 years old.

Figure 1 illustrates the comparison of the expected original norm-referenced MS risk classification with the observed Danish sample expressed in percentages. A significant chi-square was found for both the aiming and catching component score (x^2^ = 19.4692, df = 4, p = 0.001) and the KTK test (x^2^ = 27.9121, df = 4, p < 0.001). According to the original norm-referenced MS risk classification, approximately 16% of children are expected to be below average (“risk” or “difficulties” scores); similarly, 16% of children are expected to be above average (“good” or “high” scores). The percentage of children in the Danish sample who were classified as *below* average versus *above* average according to the MABC-2 aiming and catching component norm-referenced score was 9% and 16%, respectively. The equivalent percentages according to the KTK norm-referenced score was 23.5% and 9%, respectively.

### Physical activity

The median (5^th^–95^th^ percentile) number of included valid days was 7 (4–8); the median hours per day were 13 (12–15) during weekdays, and 12 (11–15) during weekend days. The median (5^th^–95^th^ percentile) hours per day spent at preschool during weekdays was 7 (5–8). No differences were observed between genders.

#### Physical activity and demographic factors

PA data are presented for children who met the PA inclusion criteria (n = 386). One child with valid data was excluded due to extremely high PA values. No difference was found in the weekly mean (SD) PA level between boys and girls (818 [190] versus 785 [187], p = 0.11), or between children in the urban versus rural areas (799 [173] versus 816 [247], p = 0.46). Boys’ weekly PA level were higher than girls’ when the preschool was located in a rural area (862 [202] versus 773 [161], p = 0.02). No gender differences in weekly PA were identified across parental educational groups or in their mother’s country of birth.

#### Physical Activity Patterns

**Figure 2:**
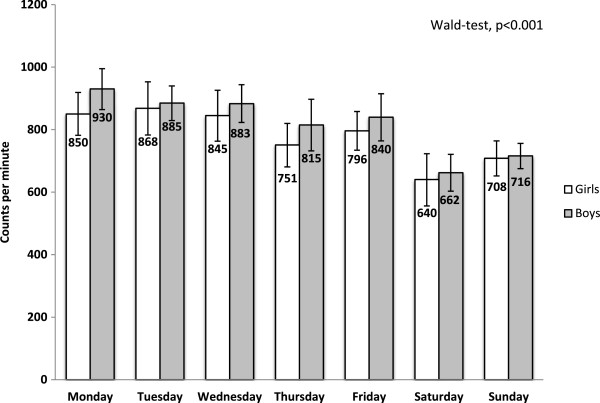
**Physical activity level across days in preschool children.** The *p*-value refers to a test of the null hypothesis that no difference in physical activity exist between days of the week. Adjustments for the country of birth of the child’s mother. Physical activity is expressed as mean counts per minute with 95% confidence interval. The illustration includes accelerometer data from 194 girls and 192 boys 5-6 years old.

Figure 2 shows that, irrespective of gender, preschool children’s PA levels vary from Monday to Sunday (Wald test, p < 0.001). A visual inspection of Figure 2 indicates a high PA level on Mondays followed by a decline over the course of the week, resulting in a remarkably lower PA level during weekend days.

**Figure 3:**
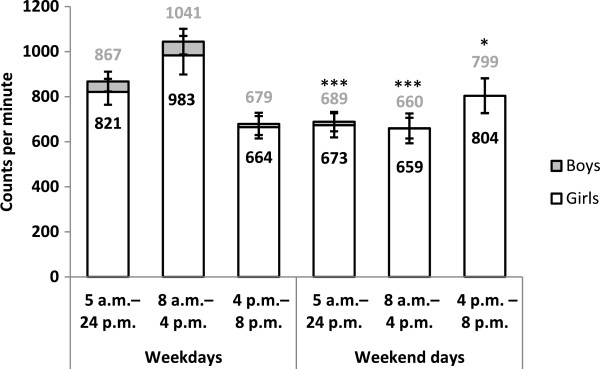
**Physical activity during defined time periods and by day type in preschool children.** The *p*-value refers to a test of the null hypothesis that no difference in PA exist between weekday and weekends for the entire day (5 a.m.-24 p.m.), 8 a.m.-4 p.m., and 4 p.m.-8 p.m., respectively. *p < 0.05, ***p < 0.001. Adjustments for country of birth of the child’s mother. Physical activity is expressed as mean counts per minute with 95% confidence interval. The illustration includes accelerometer data from 194 girls and 192 boys 5-6 years old.

Figure 3 illustrates the children’s PA levels during the entire day, between 8 a.m. and 4 p.m., and from 4 p.m. until 8 p.m. by type of day and gender, respectively. Regardless of gender, the preschool children’s PA levels were 18–20% higher during weekdays than on weekend days (p < 0.001). When comparing weekdays with weekend days during the selected time periods of the day, results show that during the daytime (8 a.m. to 4 p.m.), the PA level was 33–36% higher on weekdays than on weekend days (p < 0.001). During the late afternoon and evening hours (4–8 p.m.), PA levels were 18–21% lower on weekdays compared to weekend days (p < 0.05). No interactions were found in the tested PA patterns between genders (range of p-values: 0.11-0.76). The same analyses were carried out for the children in the lowest weekly PA quartile. The PA patterns of the children in the lowest quartile were similar to the total population of children (apart from a generally lower PA level).

**Figure 4:**
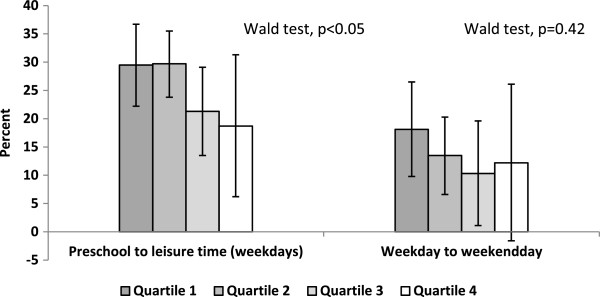
**The relative decrease (%) in mean PA between settings, and by PA quartile.** The *P*-value refers to a test of the null hypothesis that no difference in the relative decrease in mean PA exist in the transition from preschool to leisure time during weekdays, and in the transition from weekdays to weekends, respectively, between the categories of the explaining variable in question, being children in the low (quartile 1) to high PA groups (quartile 4). Preschooltime is calculated based on the preschool staff’s daily record of each child’s arrival and departure times. Adjustments for country of birth of the child’s mother. The relative decrease in mean counts per minute between the settings is expressed in percent with 95% confidence interval. The illustration includes accelerometer data from 194 girls and 192 boys 5-6 years old.

Figure 4 illustrates the relative decrease in PA from preschool attendance to leisure time, and from weekdays to weekend days, respectively, against quartiles of weekly PA. A significant difference across quartiles of weekly PA was detected with respect to the relative decrease in PA from preschool to leisure time on weekdays (Wald test, p < 0.05). No significant difference across quartiles of weekly PA was found for the transition from weekdays to weekend days.

#### Preschool effect (ICC)

Table 2 presents the between-preschool variation in total mean PA and by gender. In the model with no gender differentiation, the proportion of total variance in the mean PA level explained by the preschools was 19% (p < 0.05). However, in the gender-separated models, a marked gender effect was apparent, showing that the preschool explains a greater proportion of variance in girls (42%, p < 0.01) than in boys (6%, p = 0.13).

**Table 2:** **Preschool effect on total physical activity during preschool**

Factor	Total	Girls	Boys
	Mean PA	Mean PA	Mean PA
N	386	194	192
	Coef.(SE)	Coef.(SE)	Coef.(SE)
*Fixed part*			
Intercept	1094(33)***	1042(49)***	1132(29)***
*Random part*			
Total (n = 43)			
Between preschool variance	23837(12298)	57255(32946)	6594(4883)
Total variance	127298(21055)	136172(43497)	115920(20877)
ICC (95% CI)	0.19(0.03-34)*	0.42(0.14-0.70)**	0.06(-0.01-0.13)

*p < 0.05 **p < 0.01 ***p ≤ 0.001. Only 41 preschools are included when data are separated by gender.

## Discussion

This is the first population-based study in Denmark that uses objective measures to describe the prevalence of overweight, MS performance and objectively measured PA patterns in a large sample of Danish preschool children.

In general, no gender differences were found in the prevalence of overweight or MS performance, or in the investigated PA levels or patterns. However, gender differences were found in the MS subtests, and a higher between-preschool variation was found for girls than boys (42% versus 6%). Using the cut-points proposed by Cole et al. [25, 26], our study showed that the prevalence of being categorized as underweight, overweight or obese were 8%, 9% and 2%, respectively. According to the norm-referenced MS risk classification (Difficulties, Risk, Normal, Good, High), the proportion of children suspected of being at-risk or having motor coordination difficulties were higher than expected for motor coordination (23.5% versus 16%), but lower for the aiming and catching performance (9% versus 16%). Finally, day type and daytime comparisons showed that the children are most physically active on weekdays, during preschool time on weekdays and in the late afternoon during weekend days. Overall, less active children had similar PA patterns to the total population despite having lower PA levels. However, compared to the other PA quartiles, the relative decrease in PA from preschool to weekday leisure time seemed largest among the less active groups of children.

The reported prevalence of overweight and obesity in this study supports the statement that the obesity epidemic in young children has not worsened in Denmark since 2002 [37, 38].

The finding of a lower mean KTK norm-reference score in the Danish sample compared to the original norm-referenced data (96 [15] versus 100 [15]) is in agreement with previous studies [20–24]. Similarly, the higher KTK norm-referenced score for boys compared to girls is consistent with other European studies [20–22], with the exception of Toftegaard-Stoeckel et al. [24]. Although the KTK scores were lower than the original norm-referenced scores, this study indicated that Danish preschool children’s motor coordination is similar or slightly better than that of young children in other European countries at the present point in time [20–22].

Earlier population-based studies that used similar tests have reported that girls tend to perform better in the applied test of balance [21, 23, 39], whereas boys perform better in the “one-legged jumping test” [21, 39] and aiming and catching tests [19]. Our results supports these findings, and since the gender differences during the preschool years cannot be explained by biological factors alone, it can be assumed that different socialization factors between genders might have a negative impact on the MS performance due to a reduced level of practice, especially among girls [19, 40]. If this is the case, this highlights the importance of providing a variety of movement types for both genders during the day.

According to the norm-referenced MS risk-classification categories, the Danish sample deviated most from the expected frequencies at the extreme ends of the continuum, especially regarding the KTK test. This tendency has also been observed in previous studies [20, 21, 23], and highlights the need to monitor the further development and consequences of this possibly negative trend.

A review study by Oliver et al. [41] reported that boys are more physically active than girls during their preschool years. However, in line with our finding, most earlier studies using objective methods of assessment and including a high number of participants, do not report gender differences in the PA level of preschool children [10, 42, 43].

Looking at patterns in PA, we did not observe any interactions by gender, which is consistent with results from previous studies that used objective measures of PA [42, 44], with the exception of Verbestel et al. [10]. Only a few studies have investigated variations in PA over all seven days of the week (Monday–Sunday) in preschool-aged children [42, 43], whereas a number of studies have studied differences in PA between weekends and weekdays [10, 14, 15, 42, 44–47]. Our finding of higher PA levels during weekdays compared to weekend days are in line with both a Danish preschool study [14], and a school study [48]. Previous preschool studies from other countries have primarily reported findings consistent with ours [10, 14, 45], or reported no difference between day types [15, 42, 46]. Only a few studies have reported the opposite result, i.e. a higher PA activity level during weekend days [44, 47].

Our findings that on average Danish children were most active on weekdays, during preschool attendance on weekdays and in the late afternoon during weekend days could possibly be explained by a combination of preschool organization and parental influence.

Besides the increased focus on play during preschool attendance, outdoor time is reported to increase preschool children’s PA level, but this varies between preschool systems [8]. The children in this study were reported to play outdoors for an average (SD) of 4.6 (1.0) hours per day during preschool attendance in the summer months. In fact, the PA level in this study (as well as another Danish preschool study [11]) approaches the PA levels specifically reported for outdoor time [8], and seems to be considerably higher compared to other studies reporting PA levels specifically for preschool attendance [10, 15]. Parents are also reported to influence preschool children’s PA levels [49]. However, the parents might have less time to be active with their child during the afternoon and evening hours on weekdays because they are constrained by daily routines. On the other hand, both the parent and the child might have enough surplus energy to be more physically active in the afternoon hours during weekend days.

Children with the lowest PA level had a similar PA pattern across day type and during selected time periods of the day as the total population despite a generally lower PA level. This finding is consistent with earlier studies in schoolchildren [50, 51] and preschool children [16]. However, we did detect a difference in the relative decrease in PA from preschool to weekday leisure time between weekly PA quartiles. A visual inspection of data indicated a relatively larger decrease in PA in children in the lowest PA quartile compared to the children in the highest PA quartile.

In order to provide future initiatives to reduce physical inactivity, it is important to develop a basic understanding of the variations in PA between children. The advanced Activitystat hypothesis by Wilkin et al. according to Reilly et al. [51] states that a high proportion of PA variation between children is explained by an endogenous (probably genetic) influence. However, this hypothesis is challenged by evidence suggesting a dominant environmental influence on habitual PA [52], and highlights the need for strategies and initiatives which aim at increasing PA at multiple levels. Recently, and based on observations in a preschool setting, it was found that less physically active children were less physically active while indoors, whereas no difference in the PA level between the highly and least active children was observed during outdoor preschool time [53]. Thus, besides increasing the focus on indoor activities during preschool time, our results add that the afternoon hours could be a potentially important point of focus for studies aimed at understanding differences in PA between the least and most physically active children. To pursue this subject a bit further, we carried out a post-hoc analysis across quartiles of weekly PA based on parental questionnaire reports to study participants’ rates in organized sports and having close siblings born a few years apart. The results of a Pearson’s chi-squared test comparing proportions of groups indicated that the frequency of sports participation (p < 0.07) was lower in the quartile of the least active children compared to the remaining quartiles of children. No difference was identified with respect to the prevalence of having close siblings. Thus, until more knowledge is provided on what determines PA in this group of children, there is a need for initiatives which aim to increase PA at multiple levels throughout this group.

Finally, the ICCs representing between-preschool variation in mean PA showed that the child’s preschool explained 19% of the overall variance in preschool-time PA – a finding supported by Sugiyama et al. [12]. Additionally, the gender-separated analysis in this study indicated that Danish preschool girls have relatively more similar PA levels within preschool compared to boys, who seem less affected by the preschool they attend. This finding is supported by another preschool study in which the preschool explained a higher proportion of variance in step counts during recess in girls compared to boys [54]. Thus, when promoting PA during preschool, the preschool staff should be aware that the girls’ overall PA levels might be more affected by each other or the specific preschool’s environment than the boys’ overall PA levels. Earlier studies have shown that boys are more physically active during periods of self-organized physical activity, at least in a school setting [17], and that girls need more support than boys to initiate PA [55]. Thus, girls’ PA levels might depend more on initiated or organized activities, or possibly the structure of the playground or indoor facilities. Thus it important to reproduce and look further into possible explanations for this large between-preschool variation especially identified for the girls’ physical activity levels, and to what extent these influences can be modified in the promotion of physical activity.

A key strength of the presented study was the high participation rate, and that the participants were from a population-based random sample. Furthermore, the use of only two test observers to assess the children’s MS contributes to the validity and reliability of the MS measurements in this apparently healthy group of Danish preschool children.

The present study had some limitations that should be taken into consideration. The possible selection bias, indicate that this study sample might not be fully representative for the total population of Odense with respect to weight categorization, ethnicity and parent educational level. Presenting the prevalence of movement difficulties in well-functioning preschool children based on quantitative test results has the following limitations. First of all, a single test is not enough to determine whether a child has movement difficulties [39]. Secondly, natural fluctuations in performance during typical infants’ and preschool children’s development cannot be accounted for when applying quantitative tests that use norm-referenced data in expectation of stable development patterns [56]. Finally, the use of standardized data unadjusted to Danish children is a weakness, but nevertheless necessary for comparing the results with other studies, as well as over time.

There are well-known limitations when applying uniaxial (vertical) accelerometers in the study of physical activity patterns and levels. However, despite methodological limits, accelerometers are still one of the most preferred choices when measuring PA patterns in children’s daily life [41], and can provide important knowledge about children’s PA patterns over limited time periods.

## Conclusions

Results of this study could provide a valuable reference material for studies monitoring future trends in obesity, MS and PA behaviour in Denmark and other countries. The study did not reveal any significant gender difference among Danish preschool children in the prevalence of overweight, MS risk classification and weekly PA levels and patterns. Irrespective of gender, Danish preschool children perform well in aiming and catching, but, similar to other countries, poorer in motor coordination compared to norm-referenced data.

Similar patterns of PA across day type and during selected time periods of the day were observed for the total sample and the least active children during the season studied. However, a relatively larger decrease in PA from preschool to weekday leisure time was observed in children in the lowest PA quartile compared to children in the highest PA quartile. Knowledge about sources of variation in PA among preschool children is scarce and our findings need to be replicated in future studies. Moreover, a large between-preschool variation in physical activity was observed indicating that the preschool has a significant impact on the preschool children’s PA level, especially in girls. Future studies are needed in order to reproduce this finding and to identify which factors within the preschool setting that can explain this clustering behaviour.

## Abbreviations

PA: Physical activity
MS: Motor skills
BMI: Body mass index
KTK: Körperkoordination Test für Kinder
MABC-2: Movement Assessment Battery for Children (Second Edition)
ICC: Intraclass correlation coefficient
DUN: Danish educational nomenclature.
